# Relationship between behavioral inhibition and approach motivation systems (BIS/BAS) and intrinsic brain network connectivity in adult cannabis users

**DOI:** 10.1093/scan/nsab054

**Published:** 2021-05-05

**Authors:** Mackenzie B Taylor, Ryan Hammonds, Francesca M Filbey

**Affiliations:** Center for BrainHealth, School of Behavioral and Brain Sciences, The University of Texas at Dallas, Dallas, TX 75235, USA; Center for BrainHealth, School of Behavioral and Brain Sciences, The University of Texas at Dallas, Dallas, TX 75235, USA; Center for BrainHealth, School of Behavioral and Brain Sciences, The University of Texas at Dallas, Dallas, TX 75235, USA

**Keywords:** cannabis use disorder, motivation, behavioral inhibition, behavioral approach, functional connectivity

## Abstract

Dampened behavioral inhibition and overactive behavioral approach motivation systems (i.e. BIS/BAS) are associated with cannabis use disorder (CUD), although the underlying neural mechanisms of these alterations have not yet been examined. The brain’s executive control network (ECN) plays a role in decision-making and is associated with BIS/BAS. In this study, we tested the hypothesis that altered ECN resting-state functional connectivity (rsFC) underlies dysfunctional behavioral inhibition and approach motivation in cannabis users. To that end, we collected resting-state functional magnetic resonance imaging scans in 86 cannabis using adults and 59 non-using adults to examine group differences in the relationship between ECN rsFC and BIS/BAS. Our results showed that BIS was positively correlated with left ECN rsFC in cannabis users, while it was positively correlated with right ECN rsFC in non-users. There was a trend-level moderation effect of group on the association between BIS/BAS and ECN rsFC, showing a weaker association in BIS/BAS and ECN rsFC in cannabis users compared to non-users. An exploratory mediation analysis found that the severity of CUD mediated the relationship between users’ BIS scores and left ECN rsFC. These findings suggest that cannabis use may lead to dysregulation in typical ECN functional organization related to BIS/BAS.

## Introduction

Motivation mediates goal-directed behavior and is an important component of the addiction process. Specifically, an imbalance between increased drug-oriented motivation and dampened behavioral inhibition is considered to contribute toward the development and maintenance of substance use disorders (SUDs). Empirical evidence for altered motivation in SUD has been reported through behavioral assessments and cue-exposure paradigms (Musty and Kaback, 1995; Lane *et al.*, 2005; Bonn-Miller *et al.*, 2007; Filbey and DeWitt, 2012; Cousijn *et al.*, 2013; DeWitt *et al.*, 2013; Silins *et al.*, 2013). Such studies suggest that alterations in motivation may develop due to increased sensitization to the drug and its related cues resulting in behavioral biases (Cousijn *et al.*, 2015).

Gray introduced the concept of dual motivation systems—the behavioral inhibition and behavioral approach systems (BIS/BAS)—underlying motivated behavior (Gray, 1970). In this framework, BAS is believed to be related to action toward stimuli, while BIS is believed to regulate avoidance (Carver and White, 1994). BAS has been widely associated with SUDs (Franken, 2002; Zisserson and Palfai, 2007; O’Connor *et al.*, 2009; van Leeuwen *et al.*, 2011a), including cannabis use disorder (CUD) (van Leeuwen *et al.*, 2011a; Krmpotich *et al*., 2013; Wright *et al.*, 2016). For example, a previous study found that the initiation of cannabis use can be predicted by increased fun-seeking and reward responsivity components of BAS (van Leeuwen *et al.*, 2011a). BAS scores predicted substance use initiation in adolescents with low inhibitory control (Kim-Spoon *et al.*, 2016). Further, the activation of the BAS is thought to underlie the development of attentional biases, which drives the cue-elicited craving in cannabis users (Simons *et al.*, 2009; Filbey and DeWitt, 2012; Cousijn *et al.*, 2013, 2015; van Hemel-Ruiter *et al.*, 2013).

Recent studies have suggested that BIS/BAS is regulated by the brain’s executive control network (ECN). The ECN is a resting-state network (RSN) implicated in decision-making processes related to goal-directed behaviors such as maintenance of sustained attention (dorsolateral prefrontal cortex and parietal cortex) and response selection (pre-sensorimotor cortex and ventromedial prefrontal cortex). The relationship between BIS/BAS and ECN appears to be lateralized. Studies have suggested that BIS is associated with the right ECN (Sutton and Davidson, 1997; Eddington *et al.*, 2007; Shackman *et al.*, 2009; Spielberg *et al.*, 2011), while BAS is associated with the left ECN (Sutton and Davidson, 1997; Eddington *et al.*, 2007; Spielberg *et al.*, 2011; Krmpotich *et al*., 2013). In substance abuse, there are currently three studies that examined differences in ECN and its relationship with BIS/BAS alterations. These studies found greater resting-state functional connectivity (rsFC) in the left ECN in stimulant (Krmpotich *et al*., 2013; Yamamoto *et al.*, 2017) and nicotine users (Balconi *et al.*, 2014) relative to non-users, which was associated with BAS scores. In terms of BIS, only the study by Yamamoto *et al*. (2017) found that BIS scores were elevated in stimulant users compared to non-users. Thus, prior findings indicate that substance users show different levels of BIS/BAS and ECN connectivity compared to non-users. To date, this relationship in cannabis users and whether it differs from non-users have not yet been examined.

Studies have shown that risk-taking behavior moderates the relationship such that those with greater risk-taking behavior have increased associations between neural activation and BIS/BAS (Black *et al.*, 2014). Similarly, an association between BIS:BAS ratio imbalance toward BIS with greater connectivity in RSNs was found to be moderated by genetic risk for unhealthy weight (Olivo *et al.*, 2016). Taken together, given the above evidence of altered BIS/BAS and ECN FC in substance users, it would not only be important to determine whether dysregulation in ECN FC is linked to BIS/BAS alterations but also to understand how this brain–behavior association may differ in substance users relative to non-users.

In this study, we build on the current literature on the association between disordered motivation and substance use, by examining if rsFC within the ECN is a mechanism for altered approach motivation and inhibition in cannabis users relative to non-users. Based on previous research in heroin and nicotine using populations described above, we predicted that the strength of the linear relationship between BIS/BAS scores and ECN rsFC will differ between users and non-users (Krmpotich *et al*., 2013; Balconi *et al.*, 2014; Yamamoto *et al.*, 2017).

## Methods

### Participants

This study included 145 adult participants (59 non-users and 86 cannabis users) recruited from the Dallas metro area who provided informed consent to take part in a study aimed to determine the neurobiological mechanisms of CUD (Filbey *et al.*, 2016). The inclusion criteria were right-handedness, English as the primary language, absence of current or history of psychosis, traumatic brain injury and magnetic resonance imaging (MRI) contraindications (e.g. pregnancy, non-removal metallic implants and claustrophobia). The exclusion criteria were detection of other drugs of abuse via urinalysis (other than cannabis), regular tobacco use as defined by smoking more than a pack of cigarettes a month and current alcohol dependence based on the Structured Clinical Interview for DSM-IV (SCID) (First and Pincus, 2002). Cannabis users were recruited based on self-reported history of regular cannabis use with a minimum of 5000 lifetime occasions, as well as daily use over the preceding 60 days. Verification of cannabis use was conducted via quantification of Tetrahydrocannabinol (THC) metabolites (ng/ml; over creatinine) via gas chromatography/mass spectroscopy (GC/MS). The non-users were recruited based on the absence of daily cannabis use at any period in their lifetime, in addition to the absence of illicit drug use in the past 60 days. For initial confirmation of cannabis use or non-use, all participants came in for a baseline session where they underwent urinalysis and completed the behavioral measures described in the next section prior to the scanning session. Refer to Table 1 for a description of the participants’ demographics.

**Table 1.** Participants’ demographic information

Variables	Users	Non-users	*p*
Age, years	30.54 ± 7.16	29.42 ± 9.9	0.432
Intelligence Quotient (IQ)	104.2 ± 12.19	108.32 ± 13.9	0.061
Sex (female/male)	44/22	28/31	0.664
Psychological measures			
BDI score	8.27 ± 9.73	4.86 ± 4.87	0.014
BAI score	8.03 ± 8.69	4.22 ± 5.28	0.003
Substance use measures			
Years of regular cannabis use	11.11 ± 7.4	n/a	n/a
Frequency of cannabis use past 60 days	58.94 ± 5.6	n/a	n/a
Average grams of cannabis used on each occasion	2.24 ± 1.8	n/a	n/a
Frequency of cigarette use past 60 days	1.22 ± 3.94	0.37 ± 2.48	0.145
Current alcohol dependence symptom count	0.44 ± 0.98	0.14 ± 0.47	0.170

Values are expressed as mean ± *S.D.*

### Self-reported measures

We used the BIS/BAS scale (Carver and White, 1994) to measure avoidance and approach motivation in cannabis users and non-users. The 20-item questionnaire consists of one BIS scale (7 items) and three BAS subscales: drive (4 items), reward responsivity (5 items) and fun-seeking (4 items). Items in the BIS scale reflect motivation to avoid aversive stimuli such as punishment, while those in the BAS scale reflect motivation to approach rewarding stimuli. Reward responsivity items correspond to anticipation or occurrence of reward. Fun-seeking items correspond to desire for new rewards and impulsive approach to potential rewards. Drive items correspond to pursuit of desired goals.

We collected measures on lifetime and current CUD symptom using SCID. We also assessed depression using the Beck Depression Inventory (BDI; Beck *et al.*, 1961) and anxiety using the Beck Anxiety Inventory (BAI; Beck *et al.*, 1988). The BDI is a 21-item questionnaire of self-reported depression symptoms based on a 4-point Likert scale with a total score ranging from 0 to 67. The BAI is also a 21-item questionnaire of self-reported anxiety symptoms based on a 4-point Likert scale with a total score ranging from 0 to 63.

### Resting-state fMRI

MRI scans were collected using a 3T Philips whole body scanner equipped with Quasar gradient subsystem (40 mT/m amplitude, a slew rate of 220 mT/m/ms) at the University of Texas Southwestern Medical Center’s Advanced Imaging Research Center. Resting-state functional MRI (fMRI) scans were collected using a gradient echo, echo-planar sequence with the intercomissural line (AC–PC) as a reference (Repetition Time (TR): 2.0 s, Echo Time (TE): 29 ms, flip angle: 75°, matrix size: 64 × 64, 39 slices, voxel size: 3.44 × 3.44 × 3.5 mm^3^). Scans were collected while the participants were told to close their eyes for 5 min and think about nothing in particular. High-resolution structural scans were collected using an MPRAGE sequence (TR/TE/Inversion Time (TI): 8.2/3.70/1100 ms, flip angle: 12°, FOV: 256 × 256 mm, slab thickness: 160 mm along left-right direction, voxel size: 1 × 1 × 1 mm, total scan time: 3 min 57 s).

During the second session, users were scanned following a 72 h abstinence from cannabis use. Self-reported abstinence was verified via reduction in THC metabolites (ng/ml; over creatinine) (via GC/MS) following the 72 h abstinence relative to baseline. Participants were also asked to abstain from alcohol for 24 h (confirmed via blood alcohol content of 0.000) and from caffeine and cigarettes for the 2 h before their scheduled scan. Only individuals with confirmed abstinence were included in this study.

### Data analyses

#### Behavioral analysis: BIS/BAS scores.

We used t-tests to examine group differences on the three BAS subscale scores (drive, fun-seeking and reward responsivity) and BIS scale scores between users and non-users.

#### rsFC analyses: pre-processing and independent component analysis (ICA).

The pre-statistical processing of rsFC data consisted of motion correction using MCFLIRT, removal of timepoints corrupted by large motion using FSLMotionOutliers, brain extraction using BET and spatial smoothing using a Gaussian kernel of full-width at half-maximum of 5 mm. To reduce very-low-frequency artifacts such as scanner drift, a high-pass filtering cut-off set at 100 s was applied. Registration to high-resolution structural and standard space images was carried out using FEAT (Woolrich *et al.*, 2001). EPI volumes were registered to the individual’s structural scan using FLIRT_BBR (Boundary-Based Registration) tool (Jenkinson *et al.*, 2002).

ICA was then performed on the pre-processed data using the MELODIC tool in FSL (Multivariate Exploratory Linear Optimized Decomposition into Independent Components), Version 3.15 part of FSL v. 6.0.0 (FMRIB’s Software Library http://fsl.fmrib.ox.ac.uk/fsl). Given our interest in determining both within- and between-network connectivity, we selected an ICA approach (vs. seed-based connectivity). Noise components were identified and regressed from the single-subject ICA results using FMRIB's ICA-based X-noiseifier (FIX), before group ICA. FIX is an automated classification algorithm that attempts to identify components as ‘good’ or ‘bad’ based on a set of training data obtained by first manually classifying a subset of participants’ components (Griffanti *et al.*, 2014; Salimi-Khorshidi *et al.*, 2014). We applied an upper threshold of 20 for noise removal based on previous literature to achieve an optimal balance between the true positive and true negative rate of the independent components classified as signal and noise (Salimi-Khorshidi *et al.*, 2014; Carone *et al.*, 2017). To account for individual differences in degrees of freedom following noise removal by FIX, we calculated the total variance of components classified as noise by FIX for each participant. An independent groups *t*-test was used to compare this value between groups.

For the group-level ICA, a single 4D data set was created by temporally concatenating the pre-processed functional data. Dimensionality of group ICA was limited to 30 independent components based on a review of the methods in the current literature (Li *et al.*, 2012; Wang and Li, 2015). The set of spatial maps from the group-average analysis was used to generate subject-specific versions of the spatial maps, and associated time series, using dual regression. First, for each subject, the group-averaged set of spatial maps was regressed (as spatial regressors in a multiple regression) into the subject’s 4D space–time data set. This results in a set of subject-specific time series, one per group-level spatial map. Next, those time series were regressed (as temporal regressors, again in a multiple regression) into the same 4D data set, resulting in a set of subject-specific spatial maps, one per group-level spatial map. Spatial maps from group ICA were first regressed into each participant’s functional data to produce subject-specific time series for each component of interest. These time series were then regressed into the same functional data to produce subject-specific spatial maps for each group-level network of interest. Dual regression consists of these two stages and was implemented using FSL (Nickerson *et al.*, 2017). Given the literature reporting lateralization effects of motivation processes in ECN (Sutton and Davidson, 1997), we extracted the right and left ECN separately using the FIND lab 90 Functional Regions of Interest (fROIs) brain atlas as masks for all subsequent analyses (Shirer *et al*., 2012). A voxel-wise multiple comparison correction using a family-wise error (FWE) rate of *p *< 0.05 was applied during Randomise permutation testing (Winkler *et al.*, 2014).

#### Correlations between BIS/BAS scores and ECN rsFC.

Following the group ICA, we used General Linear Model (GLM) to correlate BIS/BAS scores and ECN rsFC. We modeled the main effects of BIS/BAS scores and group as well as their interaction on ECN rsFC. Seven GLM models tested the correlations between BIS and BAS subscale scores (drive, fun-seeking and reward responsivity) and rsFC of the ECN in users and non-users separately. These models were separated by group (two GLMs for users and two for non-users) and by the scale being examined (separate models for BIS and the three BAS subscales). The interaction effects of rsFC of the ECN and the BIS/BAS scores between cannabis users and non-users were modeled using four additional GLMs. The first GLM was tested using individual BIS scores as covariates, while the other three GLMs were tested using individual BAS subscale scores as covariates. Statistical thresholding was applied using FSL’s Randomise permutation-testing tool (5000 permutations). Clusters were determined using threshold-free cluster enhancement and a FWE-corrected cluster significance threshold of *p* < 0.05 (Beckmann *et al.*, 2009; Nickerson *et al.*, 2017).

#### BIS:BAS ratio.

To evaluate a potential imbalance between the two motivation systems, we calculated the BIS:BAS ratio according to Schutter and colleagues (Sutton and Davidson, 1997; Schutter *et al.*, 2008): BIS:BAS = (BIS − BAS)/(BIS + BAS). In this equation, positive ratios reflect an imbalance toward BIS, while negative values reflect an imbalance toward BAS. We used an analysis of variance to examine group differences using the BIS/BAS ratio. Two GLMs tested the correlations between BIS:BAS ratio and rsFC of the ECN in users and non-users separately. The interaction effect of rsFC of the ECN and the BIS:BAS ratio between cannabis users and non-users was modeled using participants’ BIS:BAS ratio values as covariates in a third GLM. Additionally, to evaluate the relationship between the BIS:BAS ratio and cannabis use, Pearson’s correlations were performed between cannabis users’ BIS:BAS ratios and total SCID CUD symptom count. 

## Results

### BIS/BAS group differences

Cannabis users had greater BAS fun-seeking subscale scores than non-users (*p* < 0.000; Table 2). BAS reward responsivity, BAS drive and total BIS scores were not significantly different between the two groups. 

**Table 2.** BIS/BAS scores between users and non-users

BIS/BAS scores	Users	Non-users	*p*
BAS drive	12.26 ± 2.04	11.81 ± 2.47	0.242
BAS fun-seeking	12.78 ± 2.07	11.42 ± 2.37	0.000*
BAS reward	17.9 ± 1.78	17.86 ± 1.9	0.921
BIS	19.36 ± 4.09	19.98 ± 3.4	0.337
BIS:BAS ratio	−0.38 ± 0.10	−0.35 ± 0.10	0.025*

Scores from the BIS/BAS scale were compared between the two groups. Cannabis users’ BAS fun-seeking scores were greater compared to non-users and their calculated BIS:BAS ratios were more imbalanced toward BAS than BIS. Values are expressed as mean ± *S.D.*

### ICA results

**Fig. 1.**
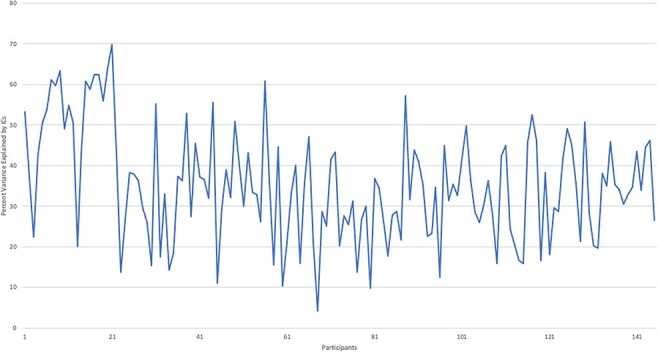
Frequency distribution of the total variance explained by participants’ ICs removed by FIX.

The total number of individual components produced for each participant prior to noise removal ranged from 38 to 56 components. The number of components from each participant that FIX classified and removed ranged from 5 to 24 components. The total variance explained by the independent components removed by FIX for each participant did not significantly differ between users (*M* = 35.14%, *S.D.* = 13.04) and non-users (*M* = 35.32%, *S.D.* = 14.60; *t *= 0.075, *p* = 0.940). Refer to Figure 1 for a frequency distribution of the variance across all participants.

### ECN rsFC group differences

There was no significant difference in ECN rsFC between users and non-users.

### Correlations between BIS/BAS and ECN rsFC

#### Users.

**Fig. 2.**
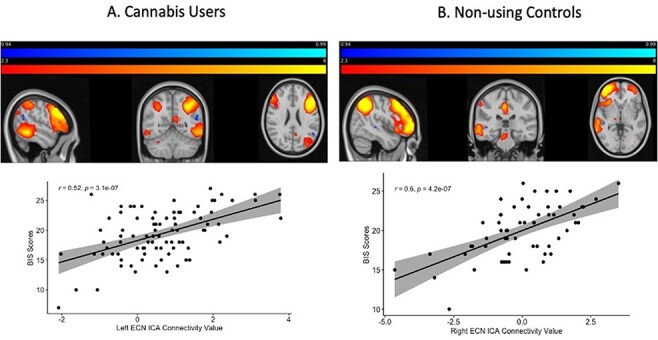
Correlation between behavioral inhibition scale scores and ECN rsFC in (A) cannabis users and (B) non-using controls.

rsFC and BIS/BAS scores revealed a significantly positive correlation between BIS scores and rsFC of the left ECN in the cannabis using group (*t* = 4.09, FWE-corrected *p *< 0.05; Figure 2, Table 3). This correlation was not significant in the right ECN. No other correlations were found between BAS subscale scores and rsFC of the ECN in the users.

**Table 3.** Correlations between BIS, BAS and FC in the ECN in cannabis users and non-users

Variable	Region, Brodmann’s area	# voxels	MNI coordinates			FWE-corrected *p*	*r*
			X	Y	Z		
BIS							
Users	Left parietal lobe, 39	22	−54	−56	24	0.039	0.528
	Left temporal lobe, 38	3	−48	18	−14	0.045	
Non-users	Right temporal lobe, 22	7	44	−30	−2	0.035	0.620
BAS reward							
Users and non-users	Left occipital lobe, 19	99	−44	−80	18	0.119	−0.351
	Right parietal lobe, 39	77	56	−52	38	0.084	
	Left occipital lobe, 19	69	0	−74	16	0.172	0.324
	Left frontal lobe, 6	20	−46	−6	28	0.146	

Reported values are for peak voxels within the left and right ECN independent components.

Areas in orange/yellow reflect the *z*-scores (color intensity reflects range of 2–8) calculated during group ICA. Areas in blue reflect the 1 − *p* value (color intensity reflects range of 0.94–0.99) obtained from the GLM. The range of *p* values was chosen for better visibility of the cluster of significant voxels. When significant voxels are found outside of the resting-state network in ICA, this indicates that the connectivity of this region with the ECN is different depending on the behavioral inhibition system (BIS) scores. The orientation of the brain images is flipped such that the right side of the figure reflects the left hemisphere of the brain and the left side of the figure reflects the right hemisphere. Scatterplots illustrate correlations between BIS scores and *z*-stat connectivity values of ECN rsFC.

#### Non-users.

rsFC and BIS scores were significantly positively correlated in the non-using group in the right ECN (*t* = 5.63, FWE-corrected *p* < 0.05; Figure 2, Table 3). This correlation was not significant in the left ECN. No other correlations were found between BAS subscale scores and rsFC of the ECN in the non-users.

#### Users vs. non-users.

Groups moderated the association between BIS/BAS and ECN rsFC at trend-level significance such that correlations between BAS reward subscale scores and left ECN rsFC (*t* = 4.52, FWE-corrected *p* = 0.08) and BIS scores and left ECN rsFC (*t* = 4.79, FWE-corrected *p* = 0.09) were greater in non-users than users.

### BIS:BAS ratio

The BIS:BAS ratios in users were more negative, showing that users had a greater imbalance toward BAS compared to non-users (Table 2, *p *= 0.025). The BIS:BAS ratio did not correlate with ECN rsFC in either group. There was no significant interaction effect between the BIS:BAS ratio and ECN rsFC in users and non-users.

As an imbalance in BIS:BAS ratio may contribute to CUD initiation and maintenance, we explored the relationship between the BIS:BAS ratio and CUD symptom count. The results showed that the BIS:BAS ratio was positively correlated with current (*r* = 0.29, *p* = 0.011) and lifetime CUD symptom count (*r* = 0.22, *p* = 0.045). 

### Post-hoc tests: mediation analyses

**Fig. 3.**
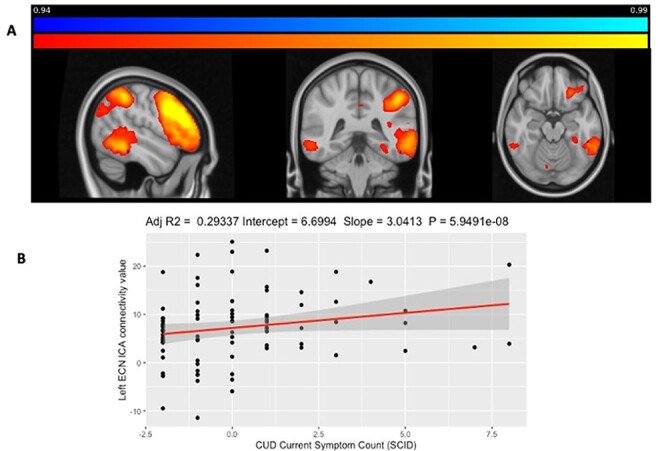
Cannabis users’ CUD symptom count correlated with left ECN rsFC.

We conducted post-hoc mediation analyses to explore the relationship between BIS, left ECN FC and CUD in the cannabis using group. We calculated Pearson’s correlations between cannabis users’ BIS scores and current SCID CUD symptom count. Then, we performed an additional GLM to correlate SCID current CUD symptom count with left ECN rsFC in cannabis users. Both were performed controlling for lifetime CUD symptom count. Controlling for lifetime symptoms reduced the potential influence of current symptoms that impact motivation. We found that cannabis users’ BIS scores were positively correlated with their SCID current CUD symptom count (*r* = 0.270, *p* = 0.018). We also found that there was a significant correlation between current CUD symptom count and left ECN rsFC (*t* = 5.79, FWE-corrected *p* = 0.046, Figure 3). Thus, the initial assumptions for the mediation analysis were met.

(A) Areas in orange/yellow reflect the *z*-scores (color intensity reflects range of 2–8) calculated during group ICA. Areas in blue reflect the 1 − *p* value (color intensity reflects range of 0.94–0.99) obtained from the GLM. The range of *p* values was chosen for better visibility of the cluster of significant voxels. The orientation of the images is flipped such that the right side of the figure reflects the left hemisphere of the brain and the left side of the figure reflects the right hemisphere. (B) Scatterplot of users’ cannabis use disorder (CUD) symptom count obtained from Structured Clinical Interview (SCID) and *z*-stat connectivity values of left ECN rsFC. Symptom count is demeaned.

**Fig. 4.**
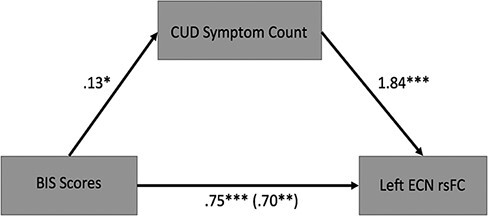
Mediation model.

We tested mediation models to determine the mediator variable and found that the effect of users’ BIS scores on their left ECN rsFC was partially mediated by current SCID CUD symptom count. The regression coefficients between BIS scores and left ECN rsFC and between left ECN rsFC and current CUD symptom count were significant. The indirect effect was (0.13) × (1.84) = 0.24. We tested the significance of this indirect effect using bootstrapping procedures. Unstandardized indirect effects were computed for each of the 1000 bootstrapped samples, and 95% confidence intervals (95% CIs) were computed. The bootstrapped unstandardized indirect effect was significant (*b* = 0.24, 95% CI [0.04, 0.48], *p* < 0.05) (Figure 4). 

For the mediation model, behavioral inhibition system (BIS) scores were the independent variable, left ECN rsFC was the dependent variable and cannabis use disorder (CUD) symptom count obtained from the Structured Clinical Interview (SCID) was the mediator. After performing a bootstrapping procedure, we found that the indirect effect was significant (*b* = 0.24, 95% CI [0.04, 0.48], *p* < 0.05). This indicates that the relationship between BIS scores and left ECN rsFC is partially mediated by CUD symptom count. *p < 0.05, **p < 0.01, ***p < 0.001.

### Manipulation check

#### Interaction between ECN and other RSNs.

Because BIS:BAS may reflect an imbalance between RSNs rather than within ECN alone, we tested the notion that BIS:BAS may be a function of the interaction between the ECN and other networks, specifically, the salience network (SN) or the default mode network (DMN). For this post-hoc analysis, we used the same methods as described above to extract the SN and the DMN from ICA results (FIND lab 90 fROIs brain atlas; Shirer *et al*., 2012). FSLNets was used to obtain the rsFC metric between the SN and ECN and between the DMN and ECN. FSLNets takes the time courses generated from the previous ICA to construct network matrices, which in this case was a 4 × 4 matrix of connection strengths between the two components identified as belonging to the SN and the two components identified as belonging to the ECN. For the DMN–ECN analysis, this resulted in a 5 × 5 matrix of connection strengths between the three components identified as belonging to the DMN and the two components identified as belonging to the ECN. Finally, a GLM was used to correlate the connection strengths within this matrix with the BIS:BAS ratios in each group and to examine any potential interaction effects of group and BIS:BAS ratios on these connection strengths. The results showed no significant correlations between SN–ECN rsFC, DMN–ECN rsFC and BIS:BAS ratios in either group. Additionally, there were no significant interaction effects of group and BIS:BAS ratios on SN–ECN rsFC or DMN–ECN rsFC.

#### ECN rsFC associations with depression and anxiety.

Because depression and anxiety have been shown to influence ECN connectivity (e.g. Stange *et al.*, 2017; Yang *et al.*, 2019), we correlated the BDI and BAI scores with ECN within-network functional connectivity. The results found no significant correlations between BDI and BAI scores and ECN rsFC in either group.

## Discussion

This study examined whether the relationship between BIS/BAS and ECN rsFC is different in cannabis users relative to non-users. Based on previous research, we predicted that the strength of the linear relationship between BIS/BAS scores and ECN rsFC will differ between users and non-users (Krmpotich *et al*., 2013; Balconi *et al.*, 2014; Yamamoto *et al.*, 2017). We found a trend-level moderation effect of group on the association between BIS/BAS and ECN rsFC, showing a weaker association in BIS/BAS and ECN rsFC in cannabis users compared to non-users (trend-level significance BAS reward = *p* < 0.08; BIS = *P* < 0.09). Because interaction effects are typically small, it is possible that the absence of a significant effect was due to statistical limitations related to the binary (*vs* continuous) classification of cannabis use (McClelland and Judd, 1993). However, these effects indicate dysregulation in typical functional networks (i.e. ECN) that underlie BIS/BAS reflected in a disrupted brain–behavior coupling in our group of cannabis users.

The notion of disrupted functional network organization in cannabis users is supported by our findings of left-lateralized effect of BIS in ECN rsFC in cannabis users, which is similar to that reported in other substance-using populations, such as in stimulant use (Davidson, 1995; Sutton and Davidson, 1997; Eddington *et al.*, 2007; Shackman *et al.*, 2009; Spielberg *et al.*, 2011; Krmpotich *et al*., 2013). The hemispheric lateralization of BIS/BAS has been attributed to associated differences in emotional valiance/motivational direction (Spielberg *et al.*, 2011). Specifically, the right hemisphere has been associated with processes important for evaluating and executing behaviors based on potential threat, a key component of BIS (Nitschke *et al.*, 2000), while the left hemisphere has been associated with response to and selection of rewards (Ramnani and Miall, 2003), which are processes related to BAS (Spielberg *et al.*, 2011). Thus, our finding suggests differential ECN lateralization with BIS in cannabis that may underlie attenuated negative reinforcement in cannabis users. 

Considering this, the current findings of lateralized effects overlap with the Integrative Cortical Unbalance Model (ICUM) in substance use, which includes the relationship between motivation systems and reward sensitivity as suggested by Finocchiaro and Balconi (2017). ICUM posits that higher activity in the left prefrontal cortex and a decrease in functional connectivity between frontal and limbic systems are related to dysfunctional reward mechanisms, including altered personality traits such as high levels of BAS and impulsivity, which reinforces compulsive behavior in addiction. This model not only suggests areas of vulnerability for developing SUDs but also incorporates underlying mechanisms contributing to the behavior characteristic of individuals with SUDs. In this manner, it implies that observed differences between users and non-users are likely due to a culmination of factors prior to and following initiation of substance use.

Consistent with previous findings, we found higher BAS fun-seeking subscale scores and a more negative BIS:BAS ratio in users compared to non-users (van Leeuwen *et al*., 2011a; Wright *et al.*, 2016). Higher BAS fun-seeking scores suggest that users have an increased valuation of fun. Additionally, an imbalance toward BAS as evidenced by more negative BIS:BAS ratios suggests that users also have reduced behavioral inhibition compared to non-users. Previous studies in other substance-using populations (e.g. alcohol, tobacco, cannabis, cocaine, methamphetamine, stimulants, heroin, ecstasy and polysubstance use) have shown that users are biased toward immediate reward compared to non-users (van Hemel-Ruiter *et al.*, 2013; Zilverstand *et al.*, 2018; Yamamoto *et al.*, 2015). Thus, these results demonstrate that cannabis users have similar imbalanced motivation systems compared to other substance users. Conversely, cannabis users did not differ from non-users on BAS reward responsivity and drive scores as we would expect based on previous research (Yamamoto *et al.*, 2017). However, van Leeuwen *et al*. (2011a) found that certain components of BAS, specifically increased fun-seeking and reward responsivity scores, can predict initiation of cannabis use in adolescents. Overall, the combined results of the van Leeuwen *et al*.’s (2011a) study in adolescents and our current study of residual cannabis use effects in regularly using adults suggest that altered components of behavioral approach contribute to cannabis use initiation and maintenance.

Although we found that BIS and BAS scores were separately related to ECN rsFC, the BIS:BAS ratio did not correlate with ECN rsFC in either group and there was no significant interaction effect of BIS:BAS ratio and ECN rsFC between groups. While the BIS:BAS imbalance suggests a bias toward behavioral approach may implicate a dysregulation between ECN and SN, post-hoc analyses did not find a correlation between SN–ECN rsFC and BIS:BAS ratio. Thus, BIS and BAS differences in cannabis users compared to non-users cannot be explained entirely by ECN rsFC. Behaviorally, we did find that BIS:BAS ratios positively correlated with symptoms related to CUD demonstrating that the greater the ratio, the greater the number of CUD-related symptoms. This finding further supports the notion of impaired motivation systems as a critical component of CUD.

Finally, our post-hoc mediation analyses suggest that BIS might promote cannabis use, perhaps through self-medication, which then leads to neurotoxic effects on higher cognitive processes (i.e. ECN FC). This suggests the possibility that dysregulation in neural networks may be remediated with the resolution of CUD. Taken together, these findings provide evidence for individual differences in the neuropsychopathology of addiction of George and Koob (2017), whereby individual differences in BIS are linked to the relationship between CUD and ECN FC, which provides avenues for the development of personalized pharmacotherapy.

Determining the underlying neural mechanisms of the BIS/BAS and their relationship to cannabis use provides important information for the advancement of treatment and prevention strategies. Specifically, differential relationships between BIS and BAS components with ECN network connectivity likely alter cannabis users’ evaluation of choices when making a decision. This altered processing of choices, as evidenced by increased BAS fun-seeking scores and an imbalance toward BAS in the BIS:BAS ratio, likely leads to risky decisions that contribute to the initiation and maintenance of cannabis use. Thus, emphasizing effective decision-making and challenging cannabis users’ propensity to value rewarding outcomes while downplaying potential consequences of their choices may help decrease relapse rates and improve poor treatment outcomes in CUD treatment (Moore and Budney, 2003).

## Limitations and conclusions

Interpretation of these findings should take into consideration that our primary finding of interaction effects was weak and only approached trend-level significance. While interaction effects often suffer from low statistical power, these findings demonstrate large correlations between BIS/BAS and rsFC in cannabis users and non-users that future studies should consider.

The implications of the current study’s results are also limited by the duration of the resting-state scan (i.e. 5 min). While there is currently no standard optimal scan duration, arguments have been made for longer resting-state fMRI scans to increase the reliability and stability of results (Birn *et al.*, 2013; Shah *et al.*, 2016). It is important to note, however, that other studies have found that estimates of correlation strength can stabilize with scan times as brief as 5 min (Van Dijk *et al.*, 2010). High test–retest reliability requires both low intra-individual variability and high inter-individual variability. The hope with longer resting-state scan durations is that by capturing more data points, the intra-individual variability can be reduced. However, ICA has been shown to be less prone to artifacts resulting from noise when compared to those determined by seed-based methods due its ability to account for structured noise effects within additional non-network components (Cole *et al.*, 2010). Additionally, a meta-analysis by Zuo and Xing (2014) identified ICA as the method with the highest test–retest reliability out of seven functional connectivity metrics and the ECN to be one of three RSNs with relatively high test–retest reliability across different methodologies. Therefore, the choice to use ICA as the functional connectivity metric and the ECN as the network of interest in this current study may counterbalance the suboptimal short scan duration. In addition, as this study only looks at brain–behavior relationships within the ECN, future studies should expand on the current findings by exploring inter-network functional connectivity, given our findings of weaker ECN and BIS/BAS correlations in users relative to non-users.

To conclude, we found implications of disrupted ECN organization that underlie BIS in cannabis users, which is partially mediated by the severity of CUD. These findings suggest that cannabis use may lead to dysregulation in typical ECN functional organization related to BIS/BAS. Dysregulation of BIS may underlie attenuated motivation to avoid harm that contributes to risky decision-making in cannabis users.

## Supplementary Material

nsab054_Supp
