# Supplementary material for: Relationship between behavioral inhibition and approach motivation systems (BIS/BAS) and intrinsic brain network connectivity in adult cannabis users
Source: Soc Cogn Affect Neurosci. 2021 May 5;16(9):985–94. doi: 10.1093/scan/nsab054 (PMC8610091; doi:10.1093/scan/nsab054)
Supplement: nsab054_Supp [file nsab054_supp.zip › nsab054_Supplementary.docx]

**Supplementary Materials**

**Reliability and validity information for questionnaire measures**

*Behavioral Inhibition system/ Behavioral Approach System* ***(****BIS/BAS)*

A study by Jorm and colleagues (1998) examined Factor structure, validity and norms in a large community sample. From the factor analyses of the BIS/BAS they found that their results largely supported the factor structure reported by Carver and White (1994). The four-factor solution corresponded to the four BIS/BAS scales, while the two-factor solution gave behavioral inhibition and behavioral activation factors. However, some limitations of the Reward Responsiveness BAS subscale were identified as it had the lowest reliability of all the BIS/BAS scales and it correlated with BIS and neuroticism as well as with the other BAS subscales. Cronbach's alpha was 0.76 for BIS, 0.83 for BAS, 0.65 for reward responsiveness, 0.80 for drive and 0.70 for fun seeking. The BIS scale correlated 0.06 with BAS, −0.05 with drive, −0.03 with fun seeking and 0.27 with reward responsiveness. Drive correlated 0.52 with fun seeking and 0.42 with reward responsiveness, while fun seeking correlated 0.45 with reward responsiveness. Results from the correlations of the BIS/BAS with other personality scales suggested that the BIS scale is largely a measure of a neuroticism/negative affectivity super-factor, while the BAS scale is a measure of an extraversion/positive affectivity super-factor. However, the BIS scale had lower correlations with measures of current anxiety and depression (r's of 0.35–0.44) than either the Neuroticism or Negative Affect scales (r's of 0.56–0.62 and 0.47–0.59, respectively), supporting the original interpretation by Carver and White (1994) that it is less a measure of experienced anxiety.

*Beck’s Depression Inventory (BDI)*

Many studies have been conducted examining the psychometric properties of the BDI. Therefore, we included information from a meta-analysis (Beck et al., 1988) that found the BDI's internal consistency estimates yielded a mean coefficient alpha of 0.86 for psychiatric patients and 0.81 for nonpsychiatric subjects. Additionally, the study found that the concurrent validitus of the BDI with respect to clinical ratings and the Hamilton Psychiatric Rating Scale for Depression (HRSD) were also high. The mean correlations of the BDI samples with clinical ratings and the HRSD were 0. 72 and 0.73, respectively, for psychiatric patients. With nonpsychiatric subjects, the mean correlations of the BDI with clinical ratings and the HRSD were 0.60 and 0.74, respectively.

*Beck’s Anxiety Inventory (BA)I*

Based on a study by Fydrich and colleagues (1992) the BAI has proved to be highly internally consistent (cronbach's alpha = .94) and acceptably reliable over an average time lapse of 11 days (r = .67). In this study, the BAI fared better on tests of convergent and discriminant validity than did Trait Anxiety. Additionally, the correlation between the BAI and Diary Anxiety was significantly higher than that between BAI and Diary Depression, and, compared to Trait Anxiety, the BAI was significantly less confounded with depression as measured by the BDI.

*Reliability Coefficients of the Current Sample*

For BIS/BAS we obtained a Cronbach's alpha of 0.747 for BIS, 0.699 for BAS Drive, 0.703 for BAS Fun Seeking, 0.676 for BAS Reward Responsiveness, and 0.798 for BAS total. These values indicate a fair level of internal consistency for the BIS/BAS scale with this specific sample. For BDI, we obtained a Cronbach's alpha of 0.926, which indicates a high level of internal consistency for the scale with this specific sample. For BAI, we obtained a Cronbach's alpha of 0.910, which indicates a high level of internal consistency for the scale with this specific sample.

**Table 1. Participant demographic information.**

Mean ± Standard Deviation

| **Variables** | **Users** | **Non-Users** | **p** |
| --- | --- | --- | --- |
| Age | 30.54±7.16 | 29.42±9.9 | .432 |
| IQ | 104.2±12.19 | 108.32±13.9 | .061 |
| Sex (F/M) | 44 / 22 | 28 / 31 | .664 |
| **Psychological Measures** |  |  |  |
| Beck Depression Inventory (BDI) Score | 8.27±9.73 | 4.86±4.87 | .014 |
| Beck Anxiety Inventory (BAI) Score | 8.03±8.69 | 4.22±5.28 | .003 |
| **Substance Use Measures** |  |  |  |
| Years of Regular Cannabis Use | 11.11± 7.4 | n/a | n/a |
| Frequency of Cannabis Use Past 60 Days | 58.94± 5.6 | n/a | n/a |
| Average Grams of Cannabis Used on Each Occasion | 2.24± 1.8 | n/a | n/a |
| Frequency of Cigarette Use Past 60 Days | 1.22±3.94 | 0.37±2.48 | *.*145 |
| Current Alcohol Dependence Symptom Count | 0.44±0.98 | 0.14±0.47 | .170 |

**Table 2. Behavioral inhibition system (BIS), behavioral approach system (BAS) scores between users and non-users.**

Scores from the Behavioral Inhibition System (BIS)/Behavioral Approach System (BAS) Scale were compared between the two groups. Cannabis Users’ BAS Fun Seeking scores were greater compared to non-users and their calculated BIS:BAS ratios were more imbalanced towards BAS than BIS. Mean ± Standard Deviation

| **BIS/BAS Scores** | **Users** | **Non-Users** | **p** |
| --- | --- | --- | --- |
| BAS Drive | 12.26± 2.04 | 11.81± 2.47 | .242 |
| BAS Fun Seeking | 12.78± 2.07 | 11.42± 2.37 | .000* |
| BAS Reward | 17.9± 1.78 | 17.86± 1.9 | .921 |
| BIS | 19.36± 4.09 | 19.98± 3.4 | .337 |
| BIS:BAS Ratio | -.38± .10 | -.35± .10 | .025* |

**Table 3. Correlations between behavioral inhibition system (BIS), behavioral approach system (BAS), and functional connectivity (FC) in the executive control network (ECN) in cannabis users and non-users.**

Reported values are for peak voxels within the left and right ECN independent components.

| **Variable** | **Region,**  **Brodmann’s area** | **# voxels** | **MNI Coordinates**  **X Y Z** | | | **FWE-corrected p** | **r** |
| --- | --- | --- | --- | --- | --- | --- | --- |
| **BIS** |  |  |  |  |  |  |  |
| Users | left parietal lobe, 39 | 22 | -54 | -56 | 24 | 0.039 | 0.528 |
|  | left temporal lobe, 38 | 3 | -48 | 18 | -14 | 0.045 |  |
| Non-Users | right temporal lobe, 22 | 7 | 44 | -30 | -2 | 0.035 | 0.620 |
| **BAS Reward** |  |  |  |  |  |  |  |
| Users  &  Non-Users | left occipital lobe, 19 | 99 | -44 | -80 | 18 | 0.119 | -0.351 |
|  | right parietal lobe, 39 | 77 | 56 | -52 | 38 | 0.084 |  |
|  | left occipital lobe, 19 | 69 | 0 | -74 | 16 | 0.172 | 0.324 |
|  | left frontal lobe, 6 | 20 | -46 | -6 | 28 | 0.146 |  |

*Supplementary Figures*

***Supplementary Figure 1.* Cannabis users’ BIS:BAS ratios correlated with BIS and BAS total scores** When comparing the relationship between cannabis users’ Behavioral Inhibition System: Behavioral Approach System (BIS:BAS) ratios and BIS versus the relationship between their BIS:BAS ratios and BAS, it appears that low BIS scores, instead of high BAS scores, largely contribute to cannabis users’ BIS:BAS ratios being imbalanced towards BAS (i.e., more negative). BAS total score is a composite of the three BAS subscale scores (drive, reward responsivity, and fun-seeking).

**
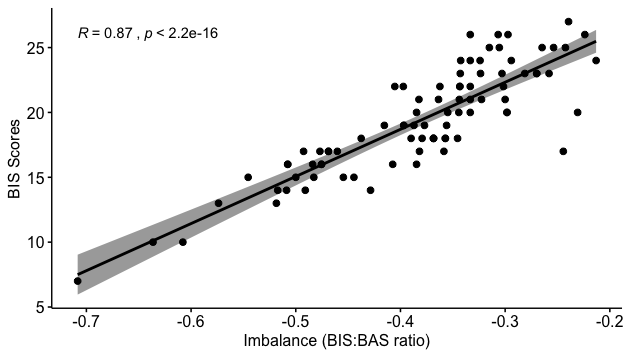

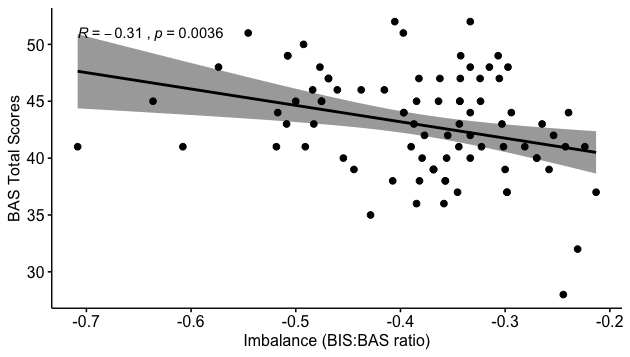
**

***Supplementary Figure 2.* Cannabis users’ BIS:BAS ratios correlated with CUD symptom count** Cannabis users’ Behavioral Inhibition System: Behavioral Approach System (BIS:BAS) ratios were correlated with their cannabis use disorder (CUD) symptom count obtained from the the Structured Clinical Interview (SCID).

**
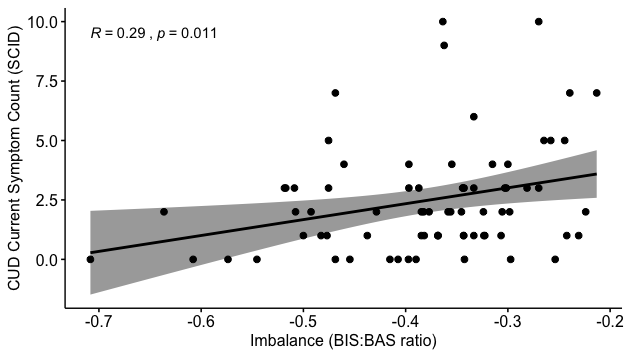

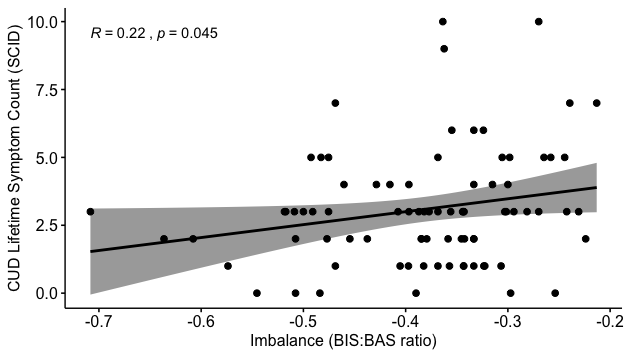
**

***Supplementary Figure 3.* Cannabis users’ BIS scores correlated with CUD symptom count**

When comparing the relationship between users’ Behavioral Inhibition System (BIS) scores and current cannabis use disorder (CUD) symptom count obtained from the Structured Clinical Interview (SCID), we found a significant positive relationship. In this figure, both scores are demeaned, as these values were entered into the mediation model with the rsFC of the left ECN.

***
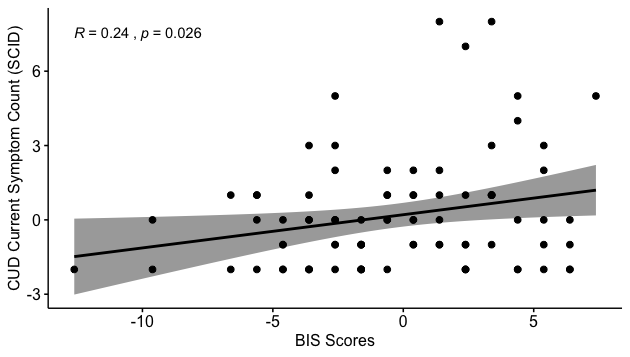
***
